# Upper Gastrointestinal Bleeding Induced by Gastric Ulcer Secondary to Strongyloidiasis: A Case Report

**DOI:** 10.31729/jnma.7924

**Published:** 2023-01-31

**Authors:** Bishal Dhakal, Sagun Dawadi, Bishnu Deep Pathak, Binit Upadhaya Regmi, Deekshanta Sitaula, Prasamsa Pudasaini, Sandesh Lamichhane, Abinash Karki, Nabin Simkhada

**Affiliations:** 1 Nepalese Army Institute of Health and Sciences, Kathmandu, Nepal; 2 Jibjibe Primary Health Care Centre, Rasuwa, Nepal; 3 Rasuwa District Hospital, Rasuwa, Nepal; 4 Chitwan Medical College, Bharatpur, Chitwan, Nepal; 5 Dhulikhel Hospital, Dhulikhel, Kavre, Nepal

**Keywords:** *gastric ulcer*, *gastrointestinal haemorrhage*, *Strongyloides stercoralis*, *strongyloidiasis*

## Abstract

Strongyloidiasis, a parasitic infestation by *Strongyloides stercoralis,* involves the gastrointestinal tract with a spectrum from duodenitis to enterocolitis. However, gastric involvement with the manifestation of upper gastrointestinal bleeding is an extremely rare condition due to *Strongyloides stercoralis*. Due to irregular excretion of larvae, unclear symptoms, paucity of effective diagnostic tools and low parasitic load, makes clinicians difficult to reach the diagnosis of strongyloidiasis. Here, we present a case of upper gastrointestinal bleeding due to a large gastric ulcer whose aetiology was identified to be *Strongyloides stercoralis* infection of the gastric region by the diagnosis of exclusion.

## INTRODUCTION

Strongyloidiasis is a helminthic infection caused by Strongyloides stercoralis. It is endemic in parts of Africa, Europe, South America and Southeast Asia with an overall prevalence of 11.7% reported in China.^1,2^ Trivial infection and the manifestation of the diseases in vulnerable populations have also been observed.^2^ Humans are chronic carriers of the parasite because of the atypical life cycle involving autoinfection which allows a repeated cycle within the human host.^3^ Alimentary tract involvement is common, however, gastric involvement of strongyloidiasis is a rare entity.^4^ Here, we present a case of strongyloidiasis manifesting as upper gastrointestinal (UGI) bleed induced by gastric ulcer.

## CASE REPORT

A 59-year-old female with no known comorbidities presented to the emergency department of our centre with the complaint of non-projectile vomiting for a period of 15 days. The vomitus was mixed with food particles and blood suggestive of hematemesis. It was also associated with loss of appetite and melena. She denied the presence of symptoms like fever, hemoptysis, diarrhoea, constipation and abdominal fullness. There was no travel history or consumption of non-steroidal anti-inflammatory drugs.

She had an ill-looking appearance on general examination with stable vital parameters. The systemic examination did not reveal any relevant findings. She was admitted for further evaluation of hematemesis. She had leukocytosis with 88% neutrophils and lymphopenia with 10% lymphocytes (Table 1).

**Table 1:** Baseline investigations at the time of admission.

Laboratory tests	Results
Total Leukocytes Count	12.5 × 10^3^/μL
Neutrophil	88%
Lymphocyte	10%
Hemoglobin	10.3 g/dl
Platelet Count	444 × 10^3^/μL
Urea	23 mg/dl
Creatinine	0.7 mg/dl
Sodium	139 mEq/L
Potassium	3.0 mEq/L
Bilirubin Total	0.6 mg/dl
Bilirubin Direct	0.3 mg/dl
Random Blood Glucose	82 mg/dl
Prothrombin time (PT)	13.5 seconds
International Normalized Ratio (INR)	1.0

**Figure 1:**
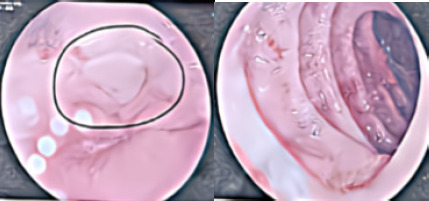
Gastroscopy showing large gastric antral ulcer with rolled out margin.

The stool occult blood test was positive and upper gastrointestinal (UGI) endoscopy showed a large ulcer with a rolled-out margin in gastric antrum (Figure 1).

To rule out the malignancy biopsy was taken from the gastric antrum and body for histopathological examination (HPE). The serological test for the human immune-deficiency virus (HIV) and hepatitis virus was non-reactive. Whilst waiting for the HPE report, differential diagnoses such as stress ulcer, HIV and portal hypertension were ruled out based on history, risk factors and laboratory investigations.

**Figure 2:**
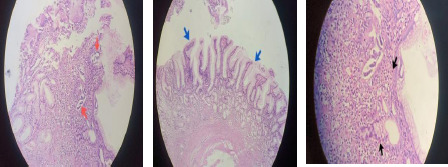
Hematoxylin-eosin-stained section of antral biopsy specimen. A) Low power field showing egg/larvae of Strongyloides within gastric mucosa, B) Low power field showing corkscrew appearance with budding of glands, C) High power field showing multiple eosinophils in lamina propria.

The HPE report showed reactive/chemical gastropathy in the background with moderate chronic active gastritis with parasitic infestation likely Strongyloides stercoralis (Figure 2). Based on Sydney classification of gastritis,^5^ it was moderately active non-atrophic antral gastritis; no intestinal metaplasia; no Helicobacter pylori. As malignancy and H. pylori evidence was ruled out from the biopsy report, Strongyloides stercoralis was considered an etiological diagnosis for upper gastrointestinal bleeding due to gastric ulcer. However, stool routine examination and polymerase chain reaction (PCR) assay of stool samples did not show eggs and larva of Strongyloides stercoralis respectively.

She was initially treated with proton pump inhibitor-pantoprazole (40 mg twice a day). After the etiological diagnosis of Strongyloides stercoralis, she was treated with ivermectin (200 mcg/kg) for 2 consecutive days. She accepted the treatment and started to regain her appetite within a week of treatment with ivermectin. She was then discharged with counselling for a follow-up within a month to assess the response to the treatment.

## DISCUSSION

Strongyloides stercoralis has been known to infect humans and non-human hosts like dogs, with the potential of infection being transmitted to and fro between the two.^6^ Strongyloides stercoralis infestation lacks recognizable eggs in stool specimens and also the larval form is very rare. Its life cycle is quite complicated as it occurs in both parasitic and free-living forms.^7^ Humans acquire the infection through penetration of the skin by the infective filariform larva. After they reach the lungs via blood circulation, they are coughed up and subsequently swallowed to reach the small intestine which is the main residence of the parasite.^8,9^ The clinical manifestations of infection due to Strongyloides stercoralis depend on the organs which are affected while the parasite completes its life cycle.

The spectrum of illness may comprise asymptomatic carrier stage, pulmonary strongyloidiasis, gastrointestinal strongyloidiasis, hyperacute syndrome, disseminated strongyloidiasis and their different manifestation and complications.^4,9-13^ Most healthy individuals with gastrointestinal strongyloidiasis may demonstrate none to minimal clinical manifestation, while others may present with anorexia, nausea, vomiting, diarrhoea, abdominal pain, and features of malnutrition.^1,11,14^ Individuals with respiratory tract involvement present with cough and individuals with immunocompromised status due to diseases like HIV, malignancy, malnutrition, and starvation as well as the immunosuppressant drugs like corticosteroids will, however, develop a more severe form of the disease.^4^ Gastric involvement in the form of a large gastric ulcer complicated with UGI bleeding in an apparently healthy individual within our case is a rare entity.

Diagnosis of strongyloidiasis is challenged by the absence of a sensitive parasitological test that can detect the organism in the stool samples.^15^ PCR-based assays for the detection of the S. stercoralis DNA as a single test for the diagnosis have improved sensitivity as compared to Baermann Concentration Test (BCT) and Agar Plate Culture (APC), two of the most sensitive stool test alone.^13,15^ Serological tests with superior sensitivity are available and may be used for screening and early detection.^13^ However, it may show cross-reactivity with other nematode antigens and prolonged positivity following the infection, thus overestimating the overall burden of the disease.^15^ This lack of a single best test for the diagnosis accentuate the need for a combination of RT-PCR and one of the parasitological test to obtain a better diagnostic yield.^15^ In our case the diagnosis of strongyloidiasis was made by the presence of the S. *stercoralis* in the biopsy taken from the edge of the ulcer, while the patient, however, reported negative for parasitological examination as well as S. *stercoralis* DNA with PCR. Depending upon the clinical presentation, different supplemental diagnostic modalities may be employed that aid and help to rule out other common diagnoses. In those with predominant UGI symptoms, endoscopy may be employed to see various manifestations of the disease which may vary from simple loss of the vascular pattern to erythema and erosions and eventually ulcers complicated by bleeding.^4,11,13^ In patients with lower GI symptoms colonoscopy may be employed to see the extent of the disease.^9^ The presence of a meagre gastric ulcer in a patient with evidence of GI strongyloidiasis however doesn't answer whether S. stercoralis is the causative agent for ulcer per se or an agent for the secondary infection of the underlying ulcer.^4^ This fact highlights the need for ruling out other causes of the ulcer, the commoner being H. Pylori infection and non-steroidal anti-inflammatory drugs (NSAIDs) use and more serious malignant ulcer by history and relevant investigations. In our case, the H. Pylori infection and the possibility of malignancy had been ruled out by the employment of gastric biopsy and the patient's denial of NSAID use. The patient also tested negative for HIV-I and HIV-II.

Ivermectin is considered the drug of choice for the treatment of strongyloidiasis and is used exclusively for the same purpose,^13^ while drugs like albendazole and other benzimidazoles though effective have a lower therapeutic potential and unfavourable side effect profile.^7^ Improvement is characterized by the resolution of symptoms, return of appetite, improvement of the nutrition profile of the patient, and resolution of any specific clinical, endoscopic or colonoscopy findings. In our case, the patient had significant improvement after treatment with Ivermectin.

In conclusion, though small bowel involvement is common in strongyloidiasis, atypical locations such as the stomach should also be considered in differentials due to the manifestations like UGI bleed as was in our case. There is still a need for the development of a newer more specific diagnostic tool for effective diagnosis of the disease and also a tool for evaluation of remission of the disease. And as strongyloidiasis is difficult to diagnose, in areas with high endemicity of the parasite, careful evaluation of history and physical findings as well as available diagnostic tool is to be carried out so that the diagnosis of the strongyloidiasis is not missed.
